# Unlocking the potential of fondaparinux: guideline for optimal usage and clinical suggestions (2023)

**DOI:** 10.3389/fphar.2024.1352982

**Published:** 2024-03-11

**Authors:** Qinan Yin, Lizhu Han, Yin Wang, Fengjiao Kang, Fengqun Cai, Liuyun Wu, Xingyue Zheng, Lian Li, Li e Dong, Limei Dong, Shuhong Liang, Min Chen, Yong Yang, Yuan Bian

**Affiliations:** ^1^ Department of Pharmacy, Sichuan Provincial People’s Hospital, University of Electronic Science and Technology of China, Chengdu, China; ^2^ Personalized Drug Therapy Key Laboratory of Sichuan Province, School of Medicine, University of Electronic Science and Technology of China, Chengdu, China; ^3^ Department of Pharmacy, The Third People’s Hospital of Chengdu, Sichuan, China; ^4^ Department of Pharmacy, the Affiliated Hospital of Southwest Medical University, Luzhou, Sichuan, China; ^5^ Department of Pharmacy, The First Affiliated Hospital of Zhengzhou University, Zhengzhou, China

**Keywords:** fondaparinux, clinical practice guideline, thromboembolic disease, clinical rational use of drugs, pharmaceutical practice, treatments

## Abstract

**Background:** Thromboembolic disease is associated with a high rate of disability or death and gravely jeopardizes people’s health and places considerable financial pressure on society. The primary treatment for thromboembolic illness is anticoagulant medication. Fondaparinux, a parenteral anticoagulant medicine, is still used but is confusing due to its disparate domestic and international indications and lack of knowledge about its usage. Its off-label drug usage in therapeutic settings and irrational drug use are also common.

**Objective:** The aim of this guideline is to enhance the judicious clinical application of fondaparinux by consolidating the findings of evidence-based research on the drug and offering superior clinical suggestions.

**Methods:** Seventeen clinical questions were developed by 37 clinical pharmacy experts, and recommendations were formulated under the supervision of three methodologists. Through methodical literature searches and the use of recommendation, assessment, development and evaluation grading techniques, we gathered evidence.

**Results:** This guideline culminated in 17 recommendations, including the use of fondaparinux for venous thromboembolism (VTE) prevention and treatment, perioperative surgical prophylaxis, specific diseases, special populations, bleeding and overdose management. For different types of VTE, we recommend first assessing thrombotic risk in hospitalized patients and then administering the drug according to the patient’s body mass. In surgical patients in the perioperative period, fondaparinux may be used for VTE prophylaxis, but postoperative use usually requires confirmation that adequate hemostasis has been achieved. Fondaparinux may be used for anticoagulation prophylaxis in patients hospitalized for oncological purposes, in patients with atrial fibrillation (AF) after resuscitation, in patients with cirrhosis combined with portal vein thrombosis (PVT), in patients with antiphospholipid syndrome (APS), and in patients with inflammatory bowel disease (IBD). Fondaparinux should be used with caution in special populations, such as pregnant female patients with a history of heparin-induced thrombocytopenia (HIT) or platelet counts less than 50 × 10^9^/L, pregnant patients with a prethrombotic state (PTS) combined with recurrent spontaneous abortion (RSA), and children. For bleeding caused by fondaparinux, dialysis may partially remove the drug.

**Conclusion:** The purpose of this guideline is to provide all healthcare providers with high-quality recommendations for the clinical use of fondaparinux and to improve the rational use of the drug in clinical practice. Currently, there is a lack of a dedicated antidote for the management of fondaparinux. The clinical investigation of activated prothrombin complex concentrate (APCC) or recombinant activated factor VII (rFⅦa) as potential reversal agents is still pending. This critical gap necessitates heightened scrutiny and research emphasis, potentially constituting a novel avenue for future inquiries into fondaparinux sodium. A meticulous examination of adverse events and safety profiles associated with the utilization of fondaparinux sodium will contribute significantly to a more comprehensive understanding of its inherent risks and benefits within the clinical milieu.

## 1 Introduction

Fondaparinux is a type of parenteral anticoagulant whose use is still confusing due to disparities in its domestic and international indications and a lack of knowledge about its use. Its off-label drug use in clinical settings and irrational drug use are also common phenomena. Encouraging the sensible use of fondaparinux in this form is especially crucial. The Chinese Medical Association Chinese Society of Clinical Pharmacy (CMACSCP) spearheaded the formation of an expert guideline on pharmaceutical practice for fondaparinux. A total of 37 clinical pharmacy experts reviewed and revised the guideline before it was finalized. Table 1 lists the recommendations for the expert guideline on fondaparinux in pharmaceutical practice.

**TABLE 1:** Summary of recommendations for the expert guideline on pharmaceutical practice for fondaparinux.

Recommendations	Recommended intensity	Quality of evidence
Recommendation 1: The Caprini score is advised for surgery, the Padua score is recommended for internal medicine, and the Khorana score is recommended for oncology patients when assessing thrombus risk in hospitalized patients. Particularly in emergency patients, fondaparinux may be used for thromboprophylaxis in hospitalized patients with an elevated risk of thrombosis and a comparatively low risk of bleeding	Strong recommendation	B
Recommendation 2: Fondaparinux can be administered during the first five to 10 days of deep vein thrombosis (DVT), particularly to patients for whom unfractionated heparin (UFH)/low molecular weight heparin (LMWH) treatment is contraindicated. For DVT treatment, the recommended daily dose is 7.5 mg, and if the patient weighs more than 100 kg, this can be increased to 10 mg; if the patient weighs less than 50 kg, the dose can be lowered to 5 mg	Strong recommendation	B
Recommendation 3: In addition to UFH and LMWH, fondaparinux may be chosen for short-term anticoagulation during the initial anticoagulation of patients with hemodynamically stable pulmonary thromboembolism (PE)	Strong recommendation	A
Recommendation 4: Fondaparinux 2.5 mg once daily, which is superior to other anticoagulant regimens, is recommended for superficial vein thrombosis (SVT) that is ≥3 cm from the deep vein junction and ≥5 cm in length. Anticoagulation should be administered for 45 days	Strong recommendation	B
Recommendation 5: SVT <3 cm from the deep venous junction and ≥5 cm in length can be treated with fondaparinux at a therapeutic dose once daily according to body weight. Regardless of whether a subsequent switch to oral anticoagulants is made, anticoagulation for 3 months is recommended before assessing the need for continued anticoagulation	Weak recommendation	D
Recommendation 6: An alternative for perioperative prevention of VTE in patients using LMWH may be fondaparinux. After evaluating surgical risk, fondaparinux normally needs to be stopped for 2–4 days before surgery in patients with regional anesthesia, and it can be resumed 6–8 h postoperatively and after catheter removal for at least 12 h (after satisfactory hemostasis) after evaluating surgical risk	Strong recommendation	B
Recommendation 7: Fondaparinux can be used to prevent perioperative VTE in individuals undergoing total knee or hip arthroplasty. Fondaparinux 2.5 mg qd is administered subcutaneously, but it cannot be started until hemostasis is established and not before 6 h following surgery (4 h after the removal of the epidural catheter)	Strong recommendation	B
Recommendation 8: Fondaparinux can be used to prevent perioperative VTE in patients undergoing hip fracture surgery (HFS). Subcutaneous fondaparinux 2.5 mg qd is recommended no earlier than 6 h after surgery (4 h after the removal of the epidural catheter) and only after hemostasis has been established	Strong recommendation	B
Recommendation 9: LMWH, fondaparinux, and UFH are the recommended parenteralanticoagulants for VTE prophylaxis in cancer inpatients	Strong recommendation	B
Recommendation 10: Fondaparinux is used for anticoagulation after cardioversion in patients with AF: 7.5 mg qd (body weight <100 kg) or 10 mg qd (body weight >100 kg) for the first week, followed by 2.5 mg qd (body weight <100 kg) or 5 mg qd (body weight >100 kg) for 3 weeks	Weak recommendation	D
Recommendation 11: In cirrhotic patients with PVT, fondaparinux is comparatively safe and effective. However, one should be mindful of the impact of cirrhosis on platelet counts	Weak recommendation	D
Recommendation 12: Patients with APS, especially those with immunogenic thrombocytopenia, may be considered for fondaparinux. After recurrent thrombosis, therapeutic anticoagulant options include vitamin K antagonists (VKAs) (international normalized ratio (INR) maintained at 2–3), LMWH, fondaparinux, or combination antiplatelet therapy on an anticoagulant basis depending on thrombosis risk	Weak recommendation	D
Recommendation 13: In patients with IBD, fondaparinux may be considered for VTE prophylaxis if the thrombosis risk is assessed as intermediate or high	Weak recommendation	D
Recommendation 14: Fondaparinux may be used with caution if anticoagulation must be considered in pregnant women with a history of HIT or a platelet count less than 50 × 10^9^/L	Weak recommendation	D
Recommendation 15: In pregnant patients with PTS combined with RSA, it is recommended to start anticoagulation after the end of menstruation in the month of preparation for pregnancy. LMWH is preferred, and fondaparinux may be used with caution if there are contraindications to its use	Weak recommendation	C
Recommendation 16: Fondaparinux can replace LMWH and warfarin for the treatment of VTE in children at a dose of 0.1 mg kg^−1^, qd	Weak recommendation	C
Recommendation 17: For bleeding due to fondaparinux, *in vitro* assays suggest that activated prothrombin complex concentrate (APCC) or recombinant activated factor VII (rFVIIa) can partially reverse the activity of fondaparinux. Dialysis partially removes fondaparinux	Strong recommendation	C

Thromboembolic disease is associated with high disability and mortality rates that seriously jeopardize people’s health. In recent years, thromboembolic disease has increasingly become a major global health problem, accounting for one quarter of deaths worldwide (Wendelboe and Raskob, 2016), and includes two main aspects: (1) VTE, mainly including PE and DVT; and (2) arterial thromboembolism, including acute coronary syndrome (ACS). VTE is a vascular disease caused by blood clotting, usually in the deep veins of the lower extremities, which can lead to serious complications such as PE. ACS, on the other hand, is a cardiovascular disease usually caused by obstruction of blood flow in the coronary arteries. Anticoagulation is an important preventive and curative measure for thromboembolic diseases. At present, the main anticoagulant drugs commonly used in clinical practice are as follows: (1) parenteral anticoagulant drugs, such as heparins, including UFH, LMWH, etc.; (2) oral anticoagulant drugs, such as VKAs and warfarin; and (3) new oral anticoagulant drugs (NOACs) (Diseases, 2018), including dabigatran, rivaroxaban, apixaban, edoxaban, etc. Anticoagulation in hospitalized patients is often a concern for physicians in terms of dosage form, and parenteral anticoagulants are often used as a prevention and treatment measure for nosocomial thromboembolic diseases given the risk of gastrointestinal bleeding with oral anticoagulants and the interaction between oral drugs (Gu et al., 2020). Fondaparinux is a selective inhibitor of activated factor X synthesized in France in 1988. Its simple pentose structure substantially increases its affinity for antithrombin (AT) and specifically binds to the activation site of AT through noncovalent bonds, resulting in rapid inhibition of factor Xa, which in turn reduces thrombin production and fibrin formation. Unlike UFH and LMWH, fondaparinux is not expected to bind to platelet factor IV and does not cross-react with plasma from patients with HIT. Compared with UFH and LMWH, fondaparinux has unique anticoagulant activity and a longer half-life (10–15 h). The indications and contraindications for the approval of fondaparinux in package inserts in China, Europe, and the United States are different, as detailed in Tables 2, 3.

**TABLE 2:** Approved indications for fondaparinux in different countries or regions.

	Indications
China NMPA	①For the prevention of venous thromboembolic events in patients undergoing significant orthopedic surgery of the lower extremities, such as hip fracture, significant knee surgery, or hip replacement
	②For the treatment of patients with unstable angina or non-ST-segment elevation myocardial infarction who are not indicated for urgent (<120 min) percutaneous transluminal coronary intervention (PCI)
	③For the treatment of patients with ST-segment elevation myocardial infarction treated with thrombolysis or no other forms of initial reperfusion therapy
Europe EMA	①For the prevention of venous thromboembolic events and adult patients undergoing major leg surgery, such as hip or knee surgery. It can also be used for adult patients at high risk of thrombosis, such as those who are undergoing abdominal surgery or who are forced to stay in bed due to an acute illness
	②To treat superficial venous thrombosis of the lower extremities
	③For the treatment of DVT or PE
	④For the treatment of unstable angina pectoris, ST-segment elevation myocardial infarction, or non-ST-segment elevation myocardial infarction in adults
US FDA	①For the prophylaxis of DVT that may lead to PE in patients undergoing HFS, including extended duration of anticoagulation prophylaxis; in patients undergoing hip or knee arthroplasty; and in patients undergoing abdominal surgery who are at risk for thromboembolic complications
	②For the treatment of acute DVT
	③For the treatment of acute PE

**TABLE 3:** Approved contraindications for fondaparinux in different countries or regions.

	Contraindications
China NMPA	①Patients known to have hypersensitivity to fondaparinux sodium or any excipient in the formulation;
	②Patients with clinically significant active bleeding;
	③Patients with acute bacterial endocarditis;
	④Patients with severe renal impairment, defined as a creatinine clearance <20 mL/min
Europe EMA	①Patients with severe renal impairment (creatinine clearance <30 mL/min);
	②Patients with body weight <50 kg;
	③Patients with active major bleeding, bacterial endocarditis;
	④patients with thrombocytopenia associated with a positive in vitro test for anti-platelet antibody in the presence of fondaparinux sodium;
	⑤patients with known hypersensitivity to fondaparinux sodium
US FDA	①Patients with severe renal impairment (creatinine clearance <30 mL/min);
	②Patients with active major bleeding;
	③Patients with bacterial endocarditis;
	④Patients with thrombocytopenia associated with a positive in vitro test for anti-platelet antibody in the ⑤presence of fondaparinux sodium;
	⑥Patients with body weight <50 kg (VTE prophylaxis only);
	⑦Patients with a history of severe hypersensitivity reactions to fondaparinux sodium (e.g., angioedema, anaphylactoid/anaphylactic reaction)

### 1.1 Scope and purpose

The purpose of this guideline is to provide all healthcare providers with high-quality recommendations for the clinical use of fondaparinux. The guideline can help healthcare providers enhance the rational clinical use of fondaparinux by providing recommendations for its use in the prevention and treatment of VTE, in perioperative surgical prophylaxis, in specific medical conditions, in special populations, and in the management of hemorrhage and overdose. It is expected that this guideline will help promote the rational use of fondaparinux worldwide. Patients and their caregivers using fondaparinux are likely to benefit from the contents of this guideline.

## 2 Methods

This expert guideline was developed in accordance with the guideline development process outlined in the World Health Organization (WHO) Guideline Development Manual (2014) (Organization, 2014). This guideline meets the requirements of the quality evaluation tool AGREE II (Brouwers et al., 2010) and complies with the criteria of the Reporting Items for Practice Guidelines in Healthcare (RIGHT) (Chen et al., 2017).

### 2.1 Presentation and identification of clinical problems

The working group for this guideline first searched relevant national and international guidelines and systematic reviews to collect the main concerns related to fondaparinux in clinical use and then sorted, analyzed and merged them to preliminarily identify 11 key clinical issues. Subsequently, two rounds of questionnaire surveys were conducted separately using the Delphi method to determine the clinical issues covered by this guideline. The experts scored each clinical issue separately using an online questionnaire survey in ascending order of importance from 1 to 5 points. The first round of questionnaires focused on the 11 clinical issues initially identified. The 11 clinical issues with significant differences in scores and newly added and/or omitted clinical issues were scored twice. A total of 64 questionnaires were received from 32 healthcare organizations in 20 provinces (autonomous regions and municipalities directly under the central government) and were collected in the first and second rounds of this guideline, and the final 12 clinical issues were included based on the importance scores of the clinical issues. The processes involved in identifying clinical issues are illustrated in Part II of the Supplementary Material.

For the final inclusion of key clinical questions, a multisource search was conducted in Chinese and English by two members of the Guideline Evidence Evaluation Group according to the principles of population, intervention, comparison, and outcome (PICO). The search databases included Medline, Embase, the Cochrane Library, Web of Science, UpToDate, Clinicaltrials.org, CBM, SinoMed, the Chinese Medical Journal Full-text Database, VIP, WanFang and CNKI; and commonly used foreign clinical guideline websites, such as the National Guideline Clearinghouse (NGC), Guideline International Network (GIN), Scottish Intercollegiate Guidelines Network (SIGN), National Institute for Clinical Excellence (NICE), and the WHO. The search date ranged from the inception date to 27 October 2023, and the languages of the studies were restricted to Chinese or English. The titles, keywords, or abstracts were searched, and the search strategy was adjusted according to the different databases. Systematic evaluations, meta-analyses, reticulated meta-analyses, original studies (including randomized controlled trials (RCTs), cohort studies, case‒control studies, etc.) and clinical guidelines were included.

This guideline uses AMSTAR (Shea et al., 2007) for methodological quality assessment of included systematic evaluations, meta-analyses, and reticulated meta-analyses; the Cochrane (Higgins et al., 2011) Risk of Bias Evaluation Tool for RCTs; the AGREE II (Brouwers et al., 2010) tool for methodological quality assessment of the guideline; the Newcastle–Ottawa Scale (NOS) (Wells et al., 2020) for cohort studies; and the NICE UK Evaluation Tool (NICE, 2020) for case series. The evaluation process was performed independently by two people, and if there was disagreement, the results were discussed together or resolved by consulting a third party. The quality of the evidence was graded using the GRADE (Guyatt et al., 2011) method and was categorized into A, B, C, and D (Table 3); the strength of the recommendation was determined according to the recommendations of the Evidence Retrieval and Evaluation in Evidence-Based Medicine (Yang et al., 2018), and the strength of the recommendation was categorized into strong and weak; high-quality evidence, a greater degree of clarity and convergence of values and preferences, and a lesser degree of cost and resource depletion were considered to indicate a strong recommendation; conversely, a weak recommendation was indicated. A definition of the quality of evidence classification is provided in Table 4.

**TABLE 4:** Evidence quality classification definitions.

Evidence quality	Definition
A	There is a high degree of confidence that the estimated effect value is close to the true effect value, and further research is unlikely to change the confidence in the estimated effect value
B	There is a moderate degree of confidence that the estimated effect value is near the true value; nevertheless, this is not a guarantee, and more research is likely to alter the estimated effect value’s level of confidence
C	There is limited confidence in the estimated effect value: the estimated effect value is likely to be very different from the true value. Further research is highly likely to change the confidence in the estimated effect value
D	There is little confidence in the estimated effect value: the estimated effect value is likely to be completely different from the true value. Any estimate of the effect value is highly uncertain

## 3 Formation of guideline recommendations

The Guideline Consensus Expert Group initially formulated recommendations on key issues for the rational clinical use of fondaparinux based on national and international evidence provided by the Guideline Evidence Evaluation Group and after considering the costs, benefits and disadvantages of the intervention. The first round of the Delphi questionnaire survey was conducted in March 2022, and 37 experts provided feedback. The recommendations that reached consensus in the first round were included, and the recommendations that did not reach consensus in the first round and the newly proposed recommendations were included in the second and third rounds. The second and third rounds of Delphi questionnaire surveys were conducted in April and May 2022, respectively, and the recommendations were further revised. In November 2023, after discussion in the expert group, ≥80% of the expert recommendations were ultimately defined as guideline recommendations by consensus. After external review and revision, 17 guideline recommendations were finalized.

### 3.1 External review of guideline recommendations

The guideline recommendations were followed by review by the Guideline External Review Group. The Guideline Consensus Expert Group discussed the feedback, revised the corresponding recommendations and submitted them to the Guideline Guidance Group for approval to determine the final recommendations.

### 3.2 Writing and updating of the guideline

After approval of the recommendations, a first draft of this guideline was written in accordance with the Reporting Items for Practice Guidelines in Healthcare and submitted to the Guideline Guidance Group for approval. The recommendations of this guideline are planned to be updated within 3 years in accordance with the methodology required by the update of the international guideline.

### 3.3 Supervision

This guideline was developed under the supervision of three methodologists.

## 4 Dissemination, implementation and evaluation

After the guideline is published, the Guideline Working Group will disseminate and promote the guideline mainly by (1) publishing it in relevant professional journals, websites and academic conferences; (2) organizing special planned meetings to promote the guideline in some provinces of China to ensure that medical service providers fully understand and correctly apply the guideline; and (3) understanding the dissemination of the guideline 1 year after its issuance and evaluating the impact of the guideline on clinical decision-making.

## 5 Results

### 5.1 Expert recommendations for the clinical application of fondaparinux

#### 5.1.1 Suggestions for the prevention and treatment of VTE with fondaparinux

Recommendation 1: The Caprini score is advised for surgery, the Padua score is recommended for internal medicine, and the Khorana score is recommended for oncology patients when assessing thrombus risk in hospitalized patients. Particularly in emergency patients, fondaparinux may be used for thromboprophylaxis in hospitalized patients with an elevated risk of thrombosis and a comparatively low risk of bleeding (Consent rate: 95.56%; Strength of recommendation: strong recommendation; Evidence Quality: B).

Most emergency patients have an increased risk of developing VTE during hospitalization and after discharge (Spyropoulos et al., 2011). For all emergency inpatients, comprehensive medical history collection and physical examination should be conducted to assess the risk of VTE. The Padua score is commonly used in internal medicine (Barbar et al., 2010), and the Caprini score is used for surgery, while the Khorana score is recommended for oncology patients (Khorana et al., 2008). The Caprini score, comprising approximately 40 distinct thrombotic risk factors, comprehensively encompasses all potential risk factors for VTE (venous thromboembolism) occurrence in both surgical and hospitalized patients. These risk factors include surgical type, disease status, personal and family history, among others. By evaluating these risk factors, patients are stratified according to their VTE risk. Each risk factor is assigned a score ranging from 1 to 5 based on its severity, and the cumulative score categorizes patients into four risk levels: low risk (0–1 points), moderate risk (2 points), high risk (3-4 points), and very high risk (≥5 points). Different risk levels warrant distinct VTE prophylactic measures (Golemi et al., 2019). Both the Padua score and Khorana score, akin to the Caprini score, serve as valuable tools for clinicians to gauge thrombotic risk in distinct clinical contexts and guide appropriate preventive measures. The ARTEMIS study was a randomized placebo-controlled trial. This study included 849 elderly acute hospitalized patients (aged >60 years) with a moderate to high risk of VTE, and compared with placebo, fondaparinux reduced the incidence of thrombosis over a one-month follow-up period; 18 (5.6%) patients in the fondaparinux group and 34 (10.5%) patients in the placebo group had a 46.7% (95% CI: 7.7%–69.3%) reduction in the relative risk (RR), and the incidence of major bleeding was lower in both groups (0.2%). There was no significant difference in major bleeding, minor bleeding, or death between the two groups (Cohen et al., 2006). In a randomized double-blind trial among 849 hospitalized patients aged 60 years and older with congestive heart failure, acute respiratory disease with chronic lung disease, acute infectious disease or inflammation, fondaparinux (425 patients) or placebo 2.5 mg (414 patients) administered subcutaneously once daily, the main efficacy outcomes were VTE detected by conventional bilateral venography and symptomatic VTE with secondary outcomes of hemorrhage and death. Follow-up was performed for 1 month. The risk of VTE was relatively reduced by 46.7% among patients treated with fondaparinux; 5 patients in the placebo group had symptomatic VTE; no thrombosis occurred in the fondaparinux group (*p* = 0.029); and 1 (0.2%) patient in each group had major bleeding. At the end of follow-up, 14 patients (3.3%) in the fondaparinux group and 25 patients (6.0%) in the placebo group had died (Cohen et al., 2006). In a randomized double-blind study, 1,049 patients who underwent selective major knee surgery were randomly assigned to receive subcutaneous injections of 2.5 mg of fondaparinux once daily or 30 mg of enoxaparin twice daily, both of which started postoperatively. The main efficacy outcome was VTE until postoperative Day 11, when DVT was detected by bilateral venography, symptomatic DVT, or symptomatic PE. The main safety outcome was major bleeding. The incidence of venous thrombosis was significantly lower in the fondaparinux group, occurring in 12.5% (45 of 361 patients) of patients in the fondaparinux group and 27.8% (101 of 363 patients) of patients in the enoxaparin group on Day 11. The frequency of major bleeding (including overt bleeding with a bleeding index of 2 or more) was greater in the fondaparinux group (*p* = 0.006), but there was no significant difference in the incidence of bleeding leading to death, reoperation, or occurrence in key organs between the two groups (Bauer et al., 2001). The results suggest that 2.5 mg of fondaparinux once daily is significantly better than 30 mg of enoxaparin twice daily for the prevention of DVT in surgical patients undergoing major knee surgery. A meta-analysis that included 13 RCTs involving 64,350 patients compared the efficacy and safety of fondaparinux and enoxaparin for thromboprophylaxis in hospitalized patients. The results showed that, compared with that in the enoxaparin group, the incidence of bleeding was significantly lower in the fondaparinux group (RR = 0.85, 95% CI = 0.81–0.88, I2 = 100%; *p* < 0.00001). In addition, the incidence of thrombosis was significantly lower in the fondaparinux group than in the enoxaparin group (RR = 0.52, 95% CI = 0.47, 0.58, *p* < 0.00001). There were no significant differences between the two groups in terms of the incidence of death, PE, stroke, or transfusion (Mahdy et al., 2022).

The 2017 Asian Venous Thromboembolism Guidelines: Updated Recommendations For Venous Thromboembolism Prevention (Liew et al., 2017) recommend the use of anticoagulants such as UFH, LMWH, fondaparinux, and NOACs for VTE prevention. The *2018* NICE guidelines: VTE in patients over 16 years of age: reducing the risk of hospital-acquired deep vein thrombosis or pulmonary embolism (National Guideline, 2018) recommend that patients with acute disease at greater risk of VTE than the risk of bleeding should be given anticoagulants for at least 7 days to prevent VTE, with LMWH being the first choice, and fondaparinux may be selected if LMWH is contraindicated. The American College of Chest Physicians (ACCP) Evidence-based Clinical Practice Guidelines for the Prevention of Venous Thromboembolism and Prevention of Thrombosis in Nonoperative Patients, 9th Edition (Kahn et al., 2012) recommend anticoagulant thromboprophylaxis with LMWH, low-dose UFH, or fondaparinux (Grade 1B) for hospitalized acute patients at increased risk of thrombosis.

Recommendation 2: Fondaparinux can be administered during the first five to 10 days of deep vein thrombosis (DVT), particularly to patients for whom unfractionated heparin (UFH)/low molecular weight heparin (LMWH) treatment is contraindicated. For DVT treatment, the recommended daily dose is 7.5 mg, and if the patient weighs more than 100 kg, this can be increased to 10 mg; if the patient weighs less than 50 kg, the dose can be lowered to 5 mg (Consent rate: 97.78%, Strength of recommendation: strong recommendation; Evidence Quality: B).

In a randomized, controlled, double-blind study among 2,205 patients with acute symptomatic DVT, during initial treatment, 7.5 mg of fondaparinux was subcutaneously injected once a day (5 mg for body weight <50 kg and 10 mg for >100 kg), or enoxaparin 1 mg kg^−1^ every 12 h for at least 5 days until the INR of the bridging VKAs was greater than 2.0; additionally, the incidence of recurrent symptomatic venous thromboembolic complications, major bleeding events and death events was recorded during initial treatment. The results showed that 43 of 1,098 (3.9%) patients treated with fondaparinux and 45 of 1,107 (4.1%) patients treated with enoxaparin had recurrent thromboembolic events. Major bleeding occurred in 1.1% of the patients in the fondaparinux group and 1.2% of the patients in the enoxaparin group. Mortality was 3.8% and 3.0%, respectively. The results indicated that fondaparinux is as safe and effective as enoxaparin in the treatment of patients with symptomatic DVT (Büller et al., 2004).

The 2021 ACCP Antithrombotic Therapy for Venous Thromboembolism (VTE) (2nd Update) recommend (Stevens et al., 2021) initial anticoagulation therapy with LMWH or fondaparinux (Grade 1B) for patients with acute DVT or PE. The *Chinese* Guidelines for the Prevention and Treatment of Thrombotic Diseases recommend that anticoagulant therapy be administered mainly based on a comprehensive assessment of thrombotic risk, bleeding risk, and imaging findings. Once acute DVT is confirmed, if there are no contraindications to anticoagulation, anticoagulation treatment should be started immediately. The commonly used parenteral anticoagulants are UFH, LMWH, and fondaparinux. The American Society of Hematology (ASH) 2018 Guidelines for the Management of Venous Thromboembolism: heparin-induced thrombocytopenia (Cuker et al., 2018) recommend the discontinuation of heparin and the initiation of fondaparinux anticoagulation for acute HIT with thrombosis (strong recommendation); fondaparinux and NOACs are both rational choices for anticoagulant use in these patients.

Recommendation 3: In addition to UFH and LMWH, fondaparinux may be chosen for short-term anticoagulation during the initial anticoagulation of patients with hemodynamically stable pulmonary thromboembolism (PE) (Consent rate: 97.83%, Strength of recommendation: strong recommendation; Evidence Quality: A).

A large RCT (Büller et al., 2003) included 2,213 patients with acute PE who were treated with subcutaneous injection of fondaparinux (given at the corresponding dose according to different body weights in the package insert) or continuous intravenous infusion of UFH to increase the activated partial thromboplastin time (APTT) to 1.5–2.5 times greater than normal for at least 5 days (APTT serves as a laboratory assay parameter for assessing the activity of the coagulation system within the body. Monitoring APTT aids in ensuring that a patient’s coagulation function remains within therapeutic ranges, thereby mitigating the risk of excessive anticoagulation (Ignjatovic, 2013).); 42 of the 1,103 patients (3.8%) with PE treated with fondaparinux had recurrent thromboembolic events, and 56 (5.0%) of the 1,110 patients with PE were treated with UFH. Major bleeding events occurred in 1.3% of patients treated with fondaparinux compared with 1.1% of patients treated with UFH. Mortality at 3 months was similar in both groups. These findings suggested that administering subcutaneous fondaparinux once daily is at least as effective and safe as administering intravenous adjusted doses of UFH during the initial treatment of patients with hemodynamically stable PE. Eighty Japanese patients with acute PE or DVT were divided into 3:1 subgroups: the subcutaneous fondaparinux group (once daily dosing; 50 kg, 5 mg; 50–100 kg, 7.5 mg; >100 kg, 10 mg) and the intravenous UFH group (intravenous bolus followed by instillation) for 5–10 days. The improvement rates were similar between the fondaparinux group and the UFH group at the end of initial treatment and follow-up, and neither group had recurrent symptomatic DVT. The improvement rates were 71.4% and 86.8% among 42 patients with PE in the fondaparinux group and 57.8% and 83.3% among 46 patients with DVT, respectively. In terms of safety, 1 case of major bleeding occurred during the initial treatment period in the fondaparinux group but not in the UFH group. For the initial treatment of patients with hemodynamically stable acute PE and DVT, once-daily subcutaneous injections of fondaparinux are comparable to the intravenous adjusted doses of UFH, and fondaparinux treatment does not require anticoagulation monitoring, which may be a potential alternative treatment for patients with acute VTE (Nakamura et al., 2011).

The *2012 ACCP* Guidelines for the Treatment and Prevention of Venous Thrombosis (Kearon et al., 2012) recommend that LMWH or fondaparinux is superior to UFH for anticoagulation in acute PE (Class I recommendation, Level B evidence). The Chinese Guidelines for the Prevention and Treatment of Thrombotic Diseases (Diseases, 2018) also mention that for highly suspected acute PE in clinical practice, parenteral anticoagulant therapy, such as UFH, LMWH, and fondaparinux, should be used while awaiting diagnostic results (2C). The choice of LMWH, UFH, fondaparinux or a loading dose of rivaroxaban (2B) is recommended for the initial anticoagulant treatment of acute PE.

Recommendation 4: Fondaparinux 2.5 mg once daily, which is superior to other anticoagulant regimens, is recommended for superficial vein thrombosis (SVT) that is ≥3 cm from the deep vein junction and ≥5 cm in length. Anticoagulation should be administered for 45 days (Consent rate: 95.56%, Strength of recommendation: strong recommendation; Evidence Quality: B).

The CALISTO trial (Decousus et al., 2010) included 3,002 patients with SVT who were randomly selected, with a distance of ≥3 cm and a length of ≥5 cm from the junction of deep veins. The patients were subcutaneously injected with 2.5 mg of fondaparinux qd or placebo for 45 days. The main efficacy outcomes included any cause or symptomatic VTE events (PE, DVT, SVT extending to the junction, SVT recurrence at 47 days); 13 of 1,502 patients in the fondaparinux group (0.9%) and 88 of 1,500 patients in the placebo group (5.9%) experienced primary endpoint events (the RR decreased, 85%; *p* < 0.01). Compared with that in the placebo group, the incidence of DVT or PE in the fondaparinux group was 85% lower (0.2% vs. 1.3%; *p* < 0.01). The incidence of serious adverse events was 0.7% in the fondaparinux group and 1.1% in the placebo group. This study showed that 2.5 mg of fondaparinux once daily for 45 days was effective in the treatment of patients with acute, symptomatic leg SVT without serious side effects. A meta-analysis (Duffett et al., 2019) showed that different treatments for SVT patients included (1) nonsteroidal anti-inflammatory drugs (including aspirin doses above 100 mg d^−1^), (2) anticoagulation treatment (oral or parenteral anticoagulant prescribed in the instructions), (3) surgical treatment (any acute surgical intervention [e.g., venous ligation or surgical resection/stripping of the affected superficial vein], and (4) observation/placebo (also including patients receiving aspirin doses ≤100 mg d^−1^ or gradient elastic stockings). The results showed that the incidence of adverse events among patients who received fondaparinux was the lowest, with 1.4 events per 100 patients during annual follow-up (95% CI = 0.5–2.8); moreover, the incidence of major bleeding in all treatment categories was low and similar. A study collected prospective observational real-life SVT data involving a total of 1,150 SVT patients who developed thrombosis-related symptoms within 3 weeks before enrollment. SVT was diagnosed by ultrasound, with an average thrombus extension range of 14.5 ± 10.7 cm and an average distance from the deep vein junction of 26.2 ± 14.8 cm. At baseline, 1,085 patients received anticoagulant therapy (92.5% as a preventive dose and 4.8% as an intermediate dose), 65.7% of whom received fondaparinux treatment and 22.8% of whom received LMWH treatment. Over time, the use rate of anticoagulants decreased, and the median treatment times for fondaparinux and LMWH were 38 days and 21 days, respectively. During the follow-up process, the incidence of thromboembolic events with fondaparinux was lower than that with other treatment regimens (the incidence with fondaparinux treatment was 4.4%, that with LMWH treatment was 9.6%, *p* = 0.017). After adjusting for the propensity score and duration of medication treatment, the absolute incidence of VTE in the fondaparinux group decreased by 5.2% compared to that in the LMWH group (Bauersachs et al., 2021).

The 2021 ACCP Antithrombotic Therapy for Venous Thromboembolism (VTE) (2nd Update) (Stevens et al., 2021) states that fondaparinux 2.5 mg qd is superior to other anticoagulant regimens (weak recommendation, low-quality evidence) for patients with SVT receiving anticoagulant therapy. The 2021 European Society For Vascular Surgery (ESVS) Clinical Practice on the Management of Venous Thrombosis states that when the SVT distance from the junction of deep veins is ≥3 cm and the length is ≥5 cm, it is recommended to administer 2.5 mg of fondaparinux qd, and an anticoagulant course is recommended for 45 days (I, B) (Kakkos et al., 2021).

Recommendation 5: SVT <3 cm from the deep venous junction and ≥5 cm in length can be treated with fondaparinux at a therapeutic dose once daily according to body weight. Regardless of whether a subsequent switch to oral anticoagulants is made, anticoagulation for 3 months is recommended before assessing the need for continued anticoagulation (Consent rate: 84.78%, Strength of recommendation: weak recommendation; Evidence quality: D).

A prospective case‒control study included 147 patients with SVT who were treated with subcutaneous injection of tinzaparin. The primary endpoint of this study was a 120-day recurrent venous thrombotic event, including DVT and/or PE. Patients were divided into Group A (98 patients) and Group B (49 patients), with Group A receiving a variable dose of tinzaparin for 60 days and Group B receiving a standard intermediate dose of tinzaparin for 90 days. Research has shown (Nikolakopoulos et al., 2018) that for patients with an SVT length ≥5 cm and a high risk of thromboembolism, such as extensive thromboembolism, recurrent thromboembolism, thromboembolism located at the thigh level, thromboembolism affecting the great saphenous vein (GSV) or small saphenous vein (SSV), thromboembolism <3 cm from the junction of deep veins, and malignant tumor-related thromboembolism, long-term or moderate doses of anticoagulation can be accepted, or prophylactic anticoagulant doses can be used 30–45 days after initial treatment; the total treatment period is 3 months.

The European Society for Vascular Surgery (ESVS) 2023 Clinical Practice Guidelines for Antithrombotic Treatment of Vascular Diseases recommend that patients with lower extremity superficial venous thrombosis ≤3 cm from the deep venous junction receive full-dose anticoagulation for 3 months to reduce the risk of further thromboembolic events (Twine et al., 2023).

#### 5.1.2 Prophylactic recommendations for fondaparinux during the perioperative period

Recommendation 6: An alternative for perioperative prevention of VTE in patients using LMWH may be fondaparinux. After evaluating surgical risk, fondaparinux normally needs to be stopped for 2–4 days before surgery in patients with regional anesthesia, and it can be resumed 6–8 h postoperatively and after catheter removal for at least 12 h (after satisfactory hemostasis) after evaluating surgical risk (Consent rate: 100%, strength of recommendation: strong recommendation; Quality of evidence: B).

A retrospective cohort study was performed to compare the efficacy and safety of LMWH and fondaparinux for the prevention of VTE in Chinese patients who underwent major orthopedic surgery or trauma. A total of 2,429 patients who underwent major orthopedic surgery or trauma and who received fondaparinux (*n* = 1,177) or LMWH (*n* = 1,252) for VTE prophylaxis were included. Fondaparinux (2.5 mg daily) was injected subcutaneously for 14 days, starting 24 h after cessation of anesthesia; LMWH (4000–4100 IU daily) was administered subcutaneously for 14 days, starting 24 h after cessation of anesthesia. Before inverse probability weighting, compared with those in the LMWH group, the in-hospital VTE (0.1% vs. 0.8%, *p* = 0.032, OR = 0.11) and minor in-hospital bleeding (17.8% vs. 26.8%, *p* < 0.001, OR = 0.59) in the fondaparinux group were lower. After inverse probability weighting, in-hospital VTE (0.1% vs. 0.8%, *p* = 0.046; weighted OR = 0.12) and minor in-hospital bleeding (17.8% vs. 26.8%, *p* < 0.001; weighted OR = 0.67) were also lower in the fondaparinux group than in the LMWH group. In addition, there were no differences in major bleeding during hospitalization, hospital death, or VTE/bleeding/death within 2 months after discharge between the fondaparinux group and the LMWH group before or after treatment (both *p* > 0.05) (Fu et al., 2022). In a randomized, double-blind, controlled study, patients were scheduled to undergo abdominal surgery under general anesthesia with 2.5 mg of fondaparinux once daily or 5000 U of dalteparin subcutaneously for 5–9 days. Fondaparinux was started 6 h after surgery. The first two doses of dalteparin 2500 U were administered 2 h before surgery and 12 h after surgery. The main outcome measures were DVT and symptoms detected by venography of both lower extremities, with a diagnosis of DVT or PE until Day 10. The main safety outcome measure was major bleeding during treatment. Among the 2,048 evaluable patients, the venous thromboembolic rate was 4.6% (47 of 1,027) in the fondaparinux group and 6.1% (62 of 1,021) in the dalteparin group. Major bleeding events occurred in 3% (49 of 1,433) of the patients in the fondaparinux group and 2% (34 of 1,425) of the patients in the dalteparin group (*p* = 0.122). The results suggested that in patients who underwent abdominal surgery and had a high risk of thrombosis, postoperative fondaparinux was at least as effective as perioperative dalteparin (Agnelli et al., 2005). A review of perioperative considerations and management of patients receiving anticoagulation also mentioned that if the patients received regional anesthesia, it was recommended to discontinue fondaparinux 3–4 days prior to regional anesthesia needle puncture or catheter manipulation. Indwelling catheters were contraindicated. Usually, the first dose is given 12 h after catheter removal (Shaikh et al., 2017). A systematic meta-analysis of the use of fondaparinux and LMWH for perioperative thromboprophylaxis for VTE (Kumar et al., 2019) revealed that the incidence of VTE events was greater in the LMWH group than in the fondaparinux group up to 15 days postsurgery, and the probability of total DVT was 0.48-fold greater in the fondaparinux group than in the LMWH group; however, the incidence of major bleeding events was greater in the fondaparinux group than in the LMWH group. Another review mentioned that fondaparinux may be more effective than LMWH in preventing VTE, but it increases the incidence of major bleeding (Dong et al., 2016).

The Interventional Procedures for Spine and Pain in Patients Treated with Antiplatelet and Anticoagulant Drugs (2nd Edition) state that due to the long half-life of fondaparinux, it is recommended to discontinue the procedure for 5 half-lives or 4 days before medium- and high-risk pain surgery. For low-risk surgery, evaluation by a treating physician is required to guide the discontinuation of fondaparinux. If a more conservative approach is required, the procedure may be delayed by 2 half-lives or 2 days. For surgeries involving patients with low or intermediate bleeding risk, anticoagulation should be resumed at 6-h intervals after surgery (Narouze et al., 2018).

Recommendation 7: Fondaparinux can be used to prevent perioperative VTE in individuals undergoing total knee or hip arthroplasty. Fondaparinux 2.5 mg qd is administered subcutaneously, but it cannot be started until hemostasis is established and not before 6 h following surgery (4 h after the removal of the epidural catheter) (Consent rate: 100%, strength of recommendation: strong recommendation; Quality of evidence: B).

In a retrospective cohort study, the ability of LMWH and fondaparinux in preventing DVT after total knee arthroplasty (TKA) was compared. The LMWH group was injected subcutaneously with LMWH calcium at half of the normal dose 4–6 h after surgery. The normal dose (once daily) was injected the next day. The fondaparinux group received subcutaneous injections of 2.5 mg (once daily) starting 6 h after surgery. The treatment duration was 2 weeks. The results suggested that both LMWH and fondaparinux were effective at preventing DVT after TKA, and there was no significant difference between the two drugs in terms of the incidence of postoperative pain, perioperative blood loss, or DVT (Yang et al., 2023). A multicenter, randomized, double-blind, placebo-controlled, parallel-group dose‒response study of fondaparinux included total hip replacement (THR) and total knee replacement (TKR) patients (Fuji et al., 2008). The TKR group included 432 patients, and the THR group included 411 patients. Patients were assigned to receive once-daily subcutaneous injections of fondaparinux (0.75, 1.5, 2.5, or 3.0 mg) or placebo. The anticoagulation time ranged from Day 2 to Days 11–15 (at least 10 days, the operation occurred on Day 1). The first dose was administered 22–26 h after surgery, before 11 p.m. on Day 2; subsequently, doses were administered from 7 to 11 a.m. on Days 3–15. The first dose on Day 2 and the second dose on Day 3 were separated by at least 12 h. For TKR, the incidence of VTE was significantly lower in all groups receiving fondaparinux than in those receiving placebo (*p* < 0.01). Similarly, compared with those of the placebo, the RRs for VTE were 47.6%, 67.4%, 75.2%, and 85.5% for 0.75, 1.5, 2.5, and 3.0 mg of fondaparinux, respectively; moreover, the incidences of major and minor bleeding were not significantly different. For THR, the incidence of VTE was significantly lower (*p* < 0.01) in the 1.5, 2.5, or 3.0 mg fondaparinux treatment groups than in the placebo group. Similarly, compared with those of the placebo, the RR reductions for VTE were 28.4%, 86.4%, 78.1%, and 57.7% for 0.75, 1.5, 2.5, and 3.0 mg of fondaparinux, respectively. The results suggested that fondaparinux was effective at preventing VTE in patients with TKR or THR without increasing the risk of bleeding or other adverse events. Another domestic meta-analysis compared the efficacy and safety of fondaparinux versus enoxaparin for the prevention of VTE after major orthopedic surgery and included 1 patient who underwent knee surgery, 1 patient who underwent HFS, and 3 patients who underwent hip arthroplasty. Fondaparinux had a better preventive effect on VTE after major orthopedic surgeries than did enoxaparin. Although the risk of major bleeding was slightly greater than that with enoxaparin, fondaparinux did not increase overall mortality (Li et al., 2013). In a multicenter cohort study in Japan, prophylaxis with fondaparinux reduced the risk of DVT in patients receiving TKA and total hip arthroplasty (THA) (Migita et al., 2014). In a meta-analysis of RCTs evaluating the efficacy and safety of various anticoagulants for the treatment of THA and TKA, 35 RCTs with a total of 53,787 patients were included. After analysis, Xa inhibitors such as fondaparinux, rivaroxaban and edoxaban have better efficacy and better safety than other anticoagulants (He et al., 2021). The summary of clinical trials and statistical analyses are illustrated in Part III of the Supplementary Material.

The Guidelines for the Prevention of Venous Thromboembolism in Major Orthopedic Surgery in China (Association and Wei, 2016) and The Guidelines for Perioperative Anesthesia Management in Elderly Patients Undergoing Knee Surgery in China (Chinese Society of Anesthesiology et al., 2020) mention that for total hip or total knee arthroplasty, 2.5 mg of fondaparinux is given subcutaneously once daily and started 6–24 h after surgery (4 h after epidural catheter removal). DVT should be routinely prevented during the TKR preoperative period, and it is recommended that treatment be started at least 10–14 days and 12 h after surgery. LMWH should be given priority, and if LMWH is contraindicated, alternative agents, including fondaparinux, direct factor Xa inhibitors (apixaban, rivaroxaban), and low-dose UFH, should be used. The 2021 Asia-Pacific Guidelines for Pharmacoprophylaxis of Venous Thrombosis in Knee and Hip Arthroplasty and Hip Fracture Surgery (Yin et al., 2022) also mention that currently widely used drugs for the prevention of VTE in Asia include aspirin, UFH, LMWH, VKAs, fondaparinux, and NOACs.

Recommendation 8: Fondaparinux can be used to prevent perioperative VTE in patients undergoing hip fracture surgery (HFS). Subcutaneous fondaparinux 2.5 mg qd is recommended no earlier than 6 h after surgery (4 h after the removal of the epidural catheter) and only after hemostasis has been established (Consent rate: 100%, strength of recommendation: strong recommendation; Quality of evidence: B).

A total of 1,711 patients with fractures of the upper third of the femur were recruited at 99 centers in 21 countries and randomly assigned to receive daily subcutaneous injections of 2.5 mg fondaparinux or the first dose of fondaparinux within 6 ± 2 h after surgery, and the second dose was administered 12 h or more after the first dose. However, if surgery was postponed until 24–48 h after admission, fondaparinux was started 12 ± 2 h before surgery or 40 mg enoxaparin daily, with the first effective dose 12 ± 2 h before surgery and a second effective dose 12–24 h after surgery for at least 5 days. The main efficacy outcome was VTE up to postoperative Day 11. The main safety outcomes were major bleeding and all-cause mortality. The follow-up period was 6 weeks. The incidence of VTE on Day 11 was 8.3% (52 of 626) in the fondaparinux group and 19.1% (119 of 624) in the enoxaparin group (*p* < 0.001). The risk reduction associated with fondaparinux was 56.4% (95% CI, 39.0%–70.3%). There was no significant difference in the incidence of death or clinically relevant bleeding between the two groups. Fondaparinux was more effective than enoxaparin in preventing VTE in patients undergoing HFS (Eriksson et al., 2001; Turpie et al., 2001).

A prospective study in Japan included 84 patients who underwent HFS, 27 patients who received 1.5 or 2.5 mg of fondaparinux qd, and 28 patients who received 2000 U of enoxaparin once or twice daily for 14 consecutive days. Twenty-nine patients were not treated. All patients underwent lower extremity ultrasonography 7 days after HFS to assess the severity of DVT. The incidence of VTE and the D-dimer level at admission and 7 and 14 days after HFS were compared, as were the incidences of adverse reactions to fondaparinux and enoxaparin. The incidence of VTE and the D-dimer level on the 7th and 14th days in the fondaparinux group were significantly lower than those in the untreated group (*p* < 0.05). D-dimer levels on Day 7 were significantly lower in the enoxaparin group than in the untreated group, but there was no significant difference in the incidence of VTE. Three adverse reactions occurred. Two patients receiving treatment with fondaparinux experienced major bleeding (defined as a postoperative hemoglobin loss greater than 2 g dl^−1^ when surgical intervention was required or compared to Day 1 on Day 7 or 14), and one patient receiving enoxaparin treatment experienced minor bleeding (defined as overt bleeding that did not meet the criteria for major bleeding). The results suggested that fondaparinux was a better drug for preventing VTE after HFS. However, patients receiving fondaparinux should be monitored for bleeding (Sasaki et al., 2011).

For HFS, according to the Chinese Guidelines for the Prevention of VTE in Major Orthopedic Surgery, patients who undergo surgery within 12 h after injury should be subcutaneously injected with 2.5 mg of fondaparinux 6–24 h after surgery. Fondaparinux has a long half-life and is not recommended for preoperative use before surgery (Association and Wei, 2016).

#### 5.1.3 Suggestions for fondaparinux for specific diseases

Recommendation 9: LMWH, fondaparinux, and UFH are the recommended parenteral anticoagulants for VTE prophylaxis in cancer inpatients (Consent rate: 100%, strength of recommendation: strong; Quality of evidence: B).

VTE is a common complication of malignancies, with clinically significant VTE occurring in up to 10% of cancer patients (Barsam et al., 2013). A multicenter, open-label, prospective observational study of venous thrombosis in Japanese patients who underwent colorectal cancer surgery included 619 patients, all of whom were treated with 1.5 or 2.5 mg of fondaparinux subcutaneously qd for 4–8 days beginning 24 h after surgery. The primary endpoint was any major bleeding event, and the secondary endpoint was any symptomatic venous thromboembolic event. Major bleeding occurred in 0.81% (95% CI = 0.3–1.9) of patients, and minor bleeding occurred in 9.5% (95% CI = 7.3–12.1) of patients, with no fatal bleeding or symptomatic VTE. The results showed that the administration of 1.5 or 2.5 mg of fondaparinux 24 h after colorectal cancer surgery was safe and effective (Hata et al., 2014). A prospective randomized controlled study of the prevention of postoperative thromboembolism in patients with urinary malignancies was conducted among a total of 298 patients (152 in the fondaparinux group and 146 in the LMWH group). Low-dose UFH 5000 U bid was given until postoperative Day 1, and fondaparinux 2.5 mg qd or LMWH 2000 U was given every 12 h until postoperative Day 5. The primary endpoint was assessed as postoperative bleeding. Bleeding occurred in 21 patients (12 in the fondaparinux group and 9 in the LMWH group). Moreover, there was no significant difference in the incidence of postoperative bleeding or other adverse events between the two groups. The results suggested that fondaparinux had good safety, supporting its prophylactic use after surgery for urinary malignancies (Hata et al., 2016).

The Guidelines for the Prevention and Treatment of Tumor-Related Venous Thromboembolism (Ma et al., 2019) recommend prophylactic anticoagulant therapy for all hospitalized patients with a diagnosis of active tumors or clinically suspected tumors and without contraindications to such treatment, which is also recommended by the National Comprehensive Cancer Network (NCCN) Clinical Practice Guidelines for Cancer-Associated Venous thromboembolic Disease (2022.V2) (Streiff et al., 2021). The prophylactic anticoagulant therapy for inpatients can be LMWH, fondaparinux or UFH. The *ASH 2021* Guidelines for the Management of Venous Thromboembolism: Prophylaxis and Treatment of Patients with Cancer recommend that thromboprophylaxis with LMWH or fondaparinux be used instead of UFH in cancer patients undergoing surgery (conditional recommendation, low level of evidence) (Lyman et al., 2021). The International Clinical Practice Guidelines for the Treatment and Prevention of Venous Thromboembolism in Patients with Cancer recommend (Farge et al., 2019) that for patients with medical cancer, LMWH or fondaparinux for prophylaxis or UFH for mortality reduction (grade 1B) be used when creatinine clearance is ≥ 30 mL min^−1^. The Prevention and Treatment of Venous Thromboembolism in Cancer Patients: Update of the American Society of Clinical Oncology (ASCO) Clinical Practice Guidelines recommendation (Mandalà et al., 2011) is that when patients have contraindications for other LMWH and NOACs, the use of fondaparinux may be considered.

Recommendation 10: Fondaparinux is used for anticoagulation after cardioversion in patients with AF: 7.5 mg qd (body weight <100 kg) or 10 mg qd (body weight >100 kg) for the first week, followed by 2.5 mg qd (body weight <100 kg) or 5 mg qd (body weight >100 kg) for 3 weeks (Consent rate: 95.65%, Strength of recommendation: weak recommendation; Quality of evidence: D).

AF is an arrhythmia involving the two upper chambers of the heart. Studies in the population of patients with resuscitated AF who have received anticoagulation are lacking, and there are currently no guideline recommendations. In a multicenter, randomized, open-label, controlled, two-parallel-group, phase II pilot study, 344 patients with AF who underwent transoesophageal echocardiography (TEE) followed by cardioversion were randomized to a fondaparinux (174 patients) or standard of care (UFH + VKAs) (170 patients) group to assess the efficacy and safety of fondaparinux versus standard of care in patients undergoing cardioversion of AF guided by echocardiography. The primary endpoints were the combined incidence of cranial nerve events, systemic thromboembolism, all-cause death, and major bleeding events. There were 3 of 174 patients (1.7%) in the fondaparinux group and 2 of 170 patients (1.2%) in the standard of care group. Thrombus disappearance was greater in the fondaparinux group (11/14; 78.6%) than in the standard of care group (7/14; 50.0%). The incidence of adverse events was similar (45.4% in the fondaparinux group and 46.5% in the standard of care group) (Cohen et al., 2015). The results suggested that fondaparinux was well tolerated in a pilot study of patients undergoing TEE-guided cardioversion, and the efficacy of this treatment was similar to that of standard therapy. Fondaparinux tended to be associated with a greater tendency toward thrombolysis.

Recommendation 11: In cirrhotic patients with PVT, fondaparinux is comparatively safe and effective. However, one should be mindful of the impact of cirrhosis on platelet counts (Consent rate: 78.26%, Strength of recommendation: weak recommendation; Quality of evidence: D).

PVT is the formation of thrombosis in the main portal vein, superior mesenteric vein, inferior mesenteric vein, or splenic vein. A total of 124 patients were included in a case-control study (Zhang et al., 2017). Patients with issues in the main branches of the portal vein, splenic vein, and superior mesenteric vein accounted for 84%, 13%, and 36%, respectively. Forty-one (33%) patients received fondaparinux, and 83 (67%) patients received LMWH. Fondaparinux-treated patients had a significantly greater probability of thrombus resolution at 36 months than patients treated with LMWH (77% vs. 51%; *p* = .001), particularly at dose reduction. Multivariate analysis revealed that treatment with fondaparinux (HR = 2.38; *p* = .002) and the use of a full dose of fondaparinux (HR = 1.78; *p* = 0.035) were independent predictors of complete portal vein recanalization. Patients treated with fondaparinux had a greater bleeding rate than patients treated with LMWH did (27% vs. 13%; *p* = 0. 06). For PVT, a review recommended the use of anticoagulation agents for thromboprophylaxis according to local regimens and recommended the use of LMWH or fondaparinux instead of UFH (Basili et al., 2018).

Recommendation 12: Patients with APS, especially those with immunogenic thrombocytopenia, may be considered for fondaparinux. After recurrent thrombosis, therapeutic anticoagulant options include vitamin K antagonists (VKAs) (international normalized ratio (INR) maintained at 2–3), LMWH, fondaparinux, or combination antiplatelet therapy on an anticoagulant basis depending on thrombosis risk (Consent rate: 93.84%, Strength of recommendation: weak recommendation; Quality of evidence: D).

Antiphospholipid syndrome (APS) is an autoimmune disorder that affects various organ systems throughout the body. It is characterized by the occurrence of thrombosis or obstetric complications in individuals who persistently carry antiphospholipid antibodies. For those individuals with antiphospholipid antibodies consistently at intermediate to high-risk levels, concomitant with systemic autoimmune diseases or other conditions posing an increased risk of thrombosis, the annual risk of initial thrombotic events may escalate to as much as 5% (Garcia and Erkan, 2018). For patients with thrombotic APS, there is a paucity of published studies, with case reports of 4 patients with refractory thrombosis on fondaparinux, 3 of whom with APS remained event free at 40 months of follow-up (Sayar et al., 2021). Another case reported two patients with APS who developed severe ischemia of both lower extremities due to arterial microthrombi and received fondaparinux and mycophenolate mofetil for long-term treatment following urgent anticoagulation, immunosuppression, and therapeutic plasma exchange. Neither patient had recurrent microthrombotic disease during a 4-year follow-up (Baron and Baron, 2019).

The Working Group of the 16th International Conference on Antiphospholipid Antibodies in 2020 reported trends in the treatment of antiphospholipid syndrome (Cohen et al., 2020) and mentioned that NOACs should not be used in patients with APS with recurrent thrombosis at standard intensity. Other treatment options may include increasing the target INR range for VKAs, standard therapeutic doses of LMWH, fondaparinux, or combination antiplatelet therapy for anticoagulation if VKAs or LMWH are inappropriate. The standard anticoagulant therapy for thrombotic APS was lifetime warfarin or alternative VKAs. Due to the lack of clear evidence, the role of NOACs in thrombotic APS has not been established and has recently been discussed in international guidelines. Other anticoagulation options included LMWH, UFH, and fondaparinux (Cohen et al., 2021).

Recommendation 13: In patients with IBD, fondaparinux may be considered for VTE prophylaxis if the thrombosis risk is assessed as intermediate or high (Consent rate: 89.13%, Strength of recommendation: weak recommendation; Quality of evidence: D).

IBD patients face an elevated risk of developing DVT or PE due to factors such as inflammation response, platelet activation, alterations in coagulation factors, and the influence of pharmacological treatments (Irving et al., 2005). In a double-blind, randomized placebo-controlled trial, the investigators included 35 centers in eight countries to investigate the efficacy and safety of the anticoagulant fondaparinux for the treatment of VTE in elderly patients with a moderate or high risk of acute hospitalization. The study included a total of 849 patients aged >60 years who were admitted to the hospital for congestive heart failure, chronic lung disease, or acute infectious or inflammatory diseases such as arthritis, connective tissue disease, or IBD and were expected to remain in bed for at least 4 days. The intervention was 2.5 mg of fondaparinux or placebo administered subcutaneously once daily for 6–14 days. The primary efficacy outcomes were VTE detected by conventional bilateral venography and symptomatic VTE on Day 15. The secondary outcomes were bleeding and death. The patients were followed up for the first month. Safety analyses (10 untreated) were available for 425 patients in the fondaparinux group and 414 patients in the placebo group. A total of 644 patients (75.9%) were included in the primary efficacy analysis. VTE was detected in 5.6% (18/321) of the fondaparinux-treated patients and 10.5% (34/323) of the placebo-treated patients, with an RR reduction of 46.7% (95% CI: 7.7%–69.3%). Symptomatic VTE occurred in 5 patients in the placebo group, and asymptomatic VTE occurred in 5 patients in the fondaparinux group (*p* = 0.029). One patient (0.2%) in each arm experienced major bleeding. At the end of follow-up, 14 (3.3%) patients in the fondaparinux group and 25 (6.0%) in the placebo group had died. Fondaparinux was effective at preventing asymptomatic and symptomatic venous thromboembolic events in elderly patients with acute medical conditions, including IBD. The frequency of major bleeding events was similar in patients treated with fondaparinux and placebo (Cohen et al., 2006).

The American Society of Hematology 2018 Guidelines for the Management of Venous Thromboembolism: prevention of Inpatient and Non-Inpatient Medical Patients mention that for emergency patients, the use of UFH, LMWH, or fondaparinux is recommended rather than no parenteral anticoagulants. Among these anticoagulants, the Panel recommended the use of LMWH or fondaparinux instead of UFH.

The International Guideline 2021: Prevention of Venous and Arterial Thrombotic Events in Patients with IBD mentions that thromboprophylaxis should be given to patients with IBD of any cause during hospitalization. LMWH or fondaparinux is stated to be superior to low-dose UFH. Preventive measures should be maintained during hospitalization. Prolonged prophylaxis after discharge should be considered only for patients with high risk factors for VTE (Olivera et al., 2021).

#### 5.1.4 Recommendations for fondaparinux in special populations

Recommendation 14: Fondaparinux may be used with caution if anticoagulation must be considered in pregnant women with a history of HIT or a platelet count less than 50 × 10^9^/L (Consent rate: 84.78%, Strength of recommendation: weak recommendation; Quality of evidence: D).

Heparin is the first-line anticoagulant for pregnant women at high risk for VTE because it does not penetrate the placenta and does not cause malformations or fetal bleeding. The actual clinical incidence of HIT is very low, and most of the evidence on HIT is from a small sample of randomized prospective studies, retrospective case‒control studies or case reports. However, anticoagulant options for pregnant women with previous HIT are limited, and few studies have been conducted. In a case report (Gerhardt et al., 2007), 2 pregnant women were treated with fondaparinux. The first case was of a 30-year-old woman who was treated with 2.5 mg of fondaparinux qd beginning at week 17 of gestation and continuing throughout pregnancy until she was 6 weeks postpartum. Fondaparinux was discontinued 24 h before cesarean section. No thrombotic events or abnormal maternal bleeding was observed, and no adverse effects were noted in the neonate. The second case was of a 35-year-old woman who was treated with 2.5 mg of fondaparinux qd beginning at week 13 of gestation, and no adverse effects were observed at 26 weeks. A retrospective study that enrolled 120 women with heparin allergy or HIT who were treated with fondaparinux prophylaxis (VTE) before, during the perinatal period, and/or for 7 days after delivery demonstrated that fondaparinux was generally well tolerated and that there were no venous thromboembolic events or elevations in liver enzyme levels during treatment. In this retrospective study, fondaparinux was found to be effective and well tolerated (Dempfle et al., 2021).

The ESC Guidelines: management of Cardiovascular Diseases in Pregnancy (2018) (Regitz-Zagrosek et al., 2011) indicate that fondaparinux has rarely been studied during pregnancy, traverses the placental barrier in small amounts, is generally harmless to the fetus, and could be used with caution during pregnancy. The Thrombosis and Haemostasis Society of Australia and New Zealand (THANZ) Guideline Statement: Diagnosis and Management of H*IT* (Joseph et al., 2019) recommends discontinuing heparin anticoagulants in the acute phase of HIT and choosing non-heparin anticoagulants, mainly bivalirudin, argatroban, fondaparinux, or NOACs. Maintenance therapy is mostly replaced by VKAs, with dalteparin in individual cases (e.g., pregnant women) and with caution in combination with fondaparinux. The Chinese Expert Guideline on Heparin-induced Thrombocytopenia (2017) (Association and Journal, 2018) states that fondaparinux may be safe in patients with a history of HIT. Despite insufficient evidence, fondaparinux is recommended for pregnant women with acute or subacute HIT.

Recommendation 15: In pregnant patients with PTS combined with RSA, it is recommended to start anticoagulation after the end of menstruation in the month of preparation for pregnancy. LMWH is preferred, and fondaparinux may be used with caution if there are contraindications to its use (Consent rate: 84.78%, Strength of recommendation: weak recommendation; Quality of evidence: C).

PTS has been shown to be strongly associated with RSA (Li, 2018). In the PTS, complications such as clotting abnormalities and impaired blood flow may adversely impact the typical fetal development within the uterus, thereby elevating the risk of miscarriage. For patients with PTS and RSA due to various etiologies, the aim of treatment is to reduce or eliminate the risk of thromboembolic events, reduce the incidence of abortion and various obstetric complications, and thereby improve pregnancy outcomes. Anticoagulant and/or antiplatelet therapy is currently considered to significantly improve pregnancy outcomes in patients with RSA due to PTS and autoimmune abnormalities. In a retrospective study (Guo et al., 2020), 300 patients with thrombophilia-prone recurrent abortion were divided into an enoxaparin-treated group (n = 220) and fondaparinux-treated group (*n* = 80); enoxaparin 100 U·kg^−1^ was administered subcutaneously q12 h, fondaparinux 2.5 mg was administered subcutaneously qd, and both were administered on the day after menstruation or on which the urine human chorionic gonadotropin (HCG) level indicated pregnancy and continued throughout pregnancy (discontinued 24 h before delivery); treatment was continued 24 h after delivery until 10 days postpartum. Preeclampsia and placental abruption did not occur in either group. There were no significant differences in the live birth rate, fetal growth restriction (FGR) rate, fetal loss in mid-term pregnancy, or recurrence of spontaneous abortion in early pregnancy between the two groups. The incidence of adverse reactions in the fondaparinux group was lower than that in the enoxaparin group. In a retrospective analysis (Zhao et al., 2021), a total of 120 patients with PTS or RSA, 68 with LMWH and 52 with fondaparinux, were included to compare pregnancy outcomes and adverse reactions between the two groups. There was no significant difference in pregnancy outcomes between patients treated with fondaparinux or LMWH for RSA due to PTS, but the incidence of adverse reactions to fondaparinux was low, and the safety of this treatment was high. Another meta-analysis included 6 eligible studies with a total of 321 patients treated with fondaparinux and 546 patients treated with LMWH. The results suggested that, compared with LMWH, fondaparinux had fewer adverse effects and similar pregnancy outcomes in patients with recurrent abortions. The aim of the study was to compare the clinical efficacy of fondaparinux with that of LMWH to provide a clinical basis for the efficacy of fondaparinux in the treatment of RSA due to PTS (Mu et al., 2023).

The Chinese Expert Guidelines for the Diagnosis and Treatment of Recurrent Abortion Complicated with Prethrombotic State 2021 (Expert Consensus Writing Group of the Professional Committee on Reproductive Immunology, 2021) recommend anticoagulant therapy as the first choice for RSA due to PTS, and LMWH alone or in combination with low-dose aspirin is recommended. Although it is not clear that fondaparinux may be used, the advantages of fondaparinux are mentioned in the text.

Recommendation 16: Fondaparinux can replace LMWH and warfarin for the treatment of VTE in children at a dose of 0.1 mg kg^−1^, qd (Consent rate: 84.78%, Strength of recommendation: weak recommendation; Quality of evidence: C).

A long-term retrospective cohort study of fondaparinux for the treatment of VTE in children (Shen et al., 2020) included 277 children (<18 years of age) with an initial dose of 0.1 mg kg^−1^ qd and a mean treatment duration of 93 days; improvement in thrombotic status occurred in 91% of patients with new thrombus during fondaparinux; and 7 patients had major bleeding, mainly those with underlying conditions that led to increased bleeding risk, indicating similar efficacy and safety to other anticoagulants in children and suggesting that this approach is a reasonable option for the treatment of VTE in children. A prospective pharmacokinetic and safety study of fondaparinux in children aged 1–18 years (Young et al., 2011) at a dose of 0.1 mg kg^−1^ qd suggested that fondaparinux was safe and effective.

Due to the lack of evidence-based medical evidence related to anticoagulant therapy in children, many recommendations in foreign guidelines mainly come from evidence-based medical evidence and clinical practice experience in adults. The United States 2015 Guidelines for the Use of Anticoagulants in Children (Law and Raffini, 2015) recommend that anticoagulants for children include UFH, LMWH, warfarin, and fondaparinux, of which fondaparinux has the advantage of being administered once daily.

#### 5.1.5 Recommendations for management of bleeding or overdose induced by fondaparinux

Recommendation 17: For bleeding due to fondaparinux, *in vitro* assays suggest that activated prothrombin complex concentrate (APCC) or recombinant activated factor VII (rFVIIa) can partially reverse the activity of fondaparinux. Dialysis partially removes fondaparinux (Consent rate: 95.65%, strength of recommendation: strong recommendation; Quality of evidence: C).

Fondaparinux has no specific reversal agent, and vitamin K and protamine do not reverse the anticoagulant effect of fondaparinux (Bijsterveld et al., 2002). A preclinical animal model has shown that APCC (APCC is the concentrate of a activated blood product containing coagulation factors IX, II, VII, and X) corrects the endogenous thrombin potential and shortens the bleeding duration (Corbonnois et al., 2013). In healthy volunteers given therapeutic doses of fondaparinux, high-dose rFVIIa (90 μg kg^−1^) partially corrected prolonged APTT, endogenous thrombin potential, and *in vivo* prothrombin activation levels (Bijsterveld et al., 2002; Bijsterveld et al., 2004). Data from studies using *in vitro* coagulation assays also suggest that activation of APCC or rFVIIa partially reverses the activity of fondaparinux and that dialysis partially removes fondaparinux (Desmurs-Clavel et al., 2009; Frontera et al., 2016).

## 6 Conclusion

The purpose of this guideline is to provide clinical pharmacists and clinicians with reference to the use of fondaparinux in treating thromboembolic diseases. Some related recommendations have not met the established standard because of the limited research results at present and need to be updated further in follow-up. This guideline is not a standard for the clinical use of fondaparinux, is intended for academic guidance and is not a legal basis. In clinical practice, the specific clinical treatment scheme varies from person to person. With the development of medical science and technology, the content of this guideline will further improve.

## 7 2023 Joint committee on pharmaceutical guidelines for fondaparinux

This guideline was developed by the Chinese Medical Association Chinese Society of Clinical Pharmacy (CMACSCP). The Society first recruited expert group members on February 27, 2022; the guideline was formally launched on March 20, 2022, and was revised and completed in 20 months. The CMACSCP was responsible for initiating and developing the guidelines, and the Chinese Center for Evidence-Based Medicine at West China Hospital of Sichuan University provided methodological and evidence support.

### 7.1 Guideline revision steering committee (Sorted by the pinyin letter of the first name)

Botao Yu General Hospital of the Western War Zone
Dasheng Dang, General Hospital of the Northern Theater Command, People's Liberation Army of China (PLA)
Feng Qiu The First Affiliated Hospital of Chongqing Medical University
Haiyan Lao Guangzhou Provincial People's Hospital
Hang Xu Nanjing Drum Tower Hospital, The Affiliated Hospital of Nanjing University Medical School
Jinhua Zhang Fujian Medical University Union Hospital
Li Li Guizhou Provincial People's Hospital
Manling Ma The First Affiliated Hospital of Harbin Medical University
Na Wang The Second Affiliated Hospital of Chongqing Medical University
Ping Zheng Nanfang Hospital of Southern Medical University
Qiang Su Nanchong Central Hospital
Qingxia Zhang Xuanwu Hospital Capital Medical University
Weiyi Feng The First Affiliated Hospital of Xi'an Jiaotong University
Wei Zhang Henan Provincial People's Hospital
Yan Mou The First Affiliated Hospital of Shandong First Medical University
Yingli Zheng Fuwai Hospital, CAMS and PUMC
Yuechuan Jia General Hospital of Ningxia Medical University
Yun Ye The Affiliated Hospital of Southwest Medical University
Zhichun Gu Renji Hospital, Shanghai Jiaotong University School of Medicine
Zhigang Zhao Beijing Tiantan Hospital of Capital Medical University

### 7.2 Expert group for guideline revision (Sorted by the pinyin letter of the first name)

Basang Lamu Tibet Autonomous Region People's Hospital
Bikui Zhang The Second Xiangya Hospital of Central South University
Daihong Guo Chinese PLA General Hospital
Enwu Long Sichuan Academy of Medical Sciences, Sichuan Provincial People's Hospital
Houwen Lin Renji Hospital, Shanghai Jiaotong University School of Medicine
Jian Zhang Xinhua Hospital Affiliated To Shanghai Jiao Tong University School of Medicine
Ling Jiang The First Affiliated Hospital of University of Science and Technology of China
Liyan Miao The First Affiliated Hospital of Soochow University
Qi Chen Guizhou Provincial People's Hospital
Rongsheng Tong Sichuan Academy of Medical Sciences, Sichuan Provincial People's Hospital
Ruichen Guo Qilu Hospital of Shandong University
Weihong Ge Nanjing Drum Tower Hospital, The Affiliated Hospital of Nanjing University Medical School
Xiaoyong Qi Hebei General Hospital
Xiao Chen The First Affiliated Hospital of Sun Yat-sen University
Xinan Wu The First Hospital of Lanzhou University
Yalin Dong The First Affiliated Hospital of Xi'an Jiaotong University
Yong Yang Sichuan Academy of Medical Sciences-Sichuan Provincial People's Hospital
Yujin Guo The First People's Hospital of Jining City
