# Supplementary material for: Unlocking the potential of fondaparinux: guideline for optimal usage and clinical suggestions (2023)
Source: Front Pharmacol. 2024 Mar 11;15:1352982. doi: 10.3389/fphar.2024.1352982 (PMC10961909; doi:10.3389/fphar.2024.1352982)
Supplement: Supplementary file 1 [file DataSheet1.docx]

**Supplementary Material**

**Part Ⅰ**

These are all the abbreviations that appear in the guideline

| Full name | Abbreviation |
| --- | --- |
| activated partial thromboplastin time | APTT |
| activated prothrombin complex concentrate | APCC |
| acute coronary syndrome | ACS |
| American College of Chest Physicians | ACCP |
| American Society of Clinical Oncology | ASCO |
| American Society of Hematology | ASH |
| antiphospholipid syndrome | APS |
| antithrombin | AT |
| atrial fibrillation | AF |
| Chinese Medical Association Chinese Society of Clinical Pharmacy | CMACSCP |
| confidence interval | CI |
| deep vein thrombosis | DVT |
| European Society for Vascular Surgery | ESVS |
| European Society of Cardiology | ESC |
| fetal growth restriction | FGR |
| great saphenous vein | GSV |
| Guideline International Network | GIN |
| hazard ratio | HR |
| heparin Induced thrombocytopenia | HIT |
| hip fracture surgery | HFS |
| human chorionic gonadotropin | HCG |
| inflammatory bowel disease | IBD |
| international normalized ratio | INR |
| low molecular weight heparin | LMWH |
| National Comprehensive Cancer Network | NCCN |
| National Guideline Clearinghouse | NGC |
| National Institute for Clinical Excellence | NICE |
| Newcastle-Ottawa Scale | NOS |
| new oral anticoagulant drugs | NOACs |
| Percutaneous Transluminal Coronary Intervention | PCI |
| population, intervention, comparison, and outcome | PICO |
| portal vein thrombosis | PVT |
| prethrombotic state | PTS |
| pulmonary thromboembolism | PE |
| randomized controlled trials | RCTs |
| recombinant activated factor VII | rFVIIa |
| recurrent spontaneous abortion | RSA |
| relative risk | RR |
| Reporting Items for Practice Guidelines in Healthcare | RIGHT |
| Scottish Intercollegiate Guidelines Network | SIGN |
| small saphenous vein | SSV |
| superficial vein thrombosis | SVT |
| Thrombosis and Haemostasis Society of Australia and New Zealand | THANZ |
| total hip arthroplasty | THA |
| total hip replacement | THR |
| total knee arthroplasty | TKA |
| total knee replacement | TKR |
| transoesophageal echocardiography | TEE |
| unfractionated heparin | UFH |
| venous thromboembolism | VTE |
| vitamin K antagonists | VKAs |
| World Health Organization | WHO |

**Part Ⅱ**

The following images present the specific process used when raising and identifying clinical issues.


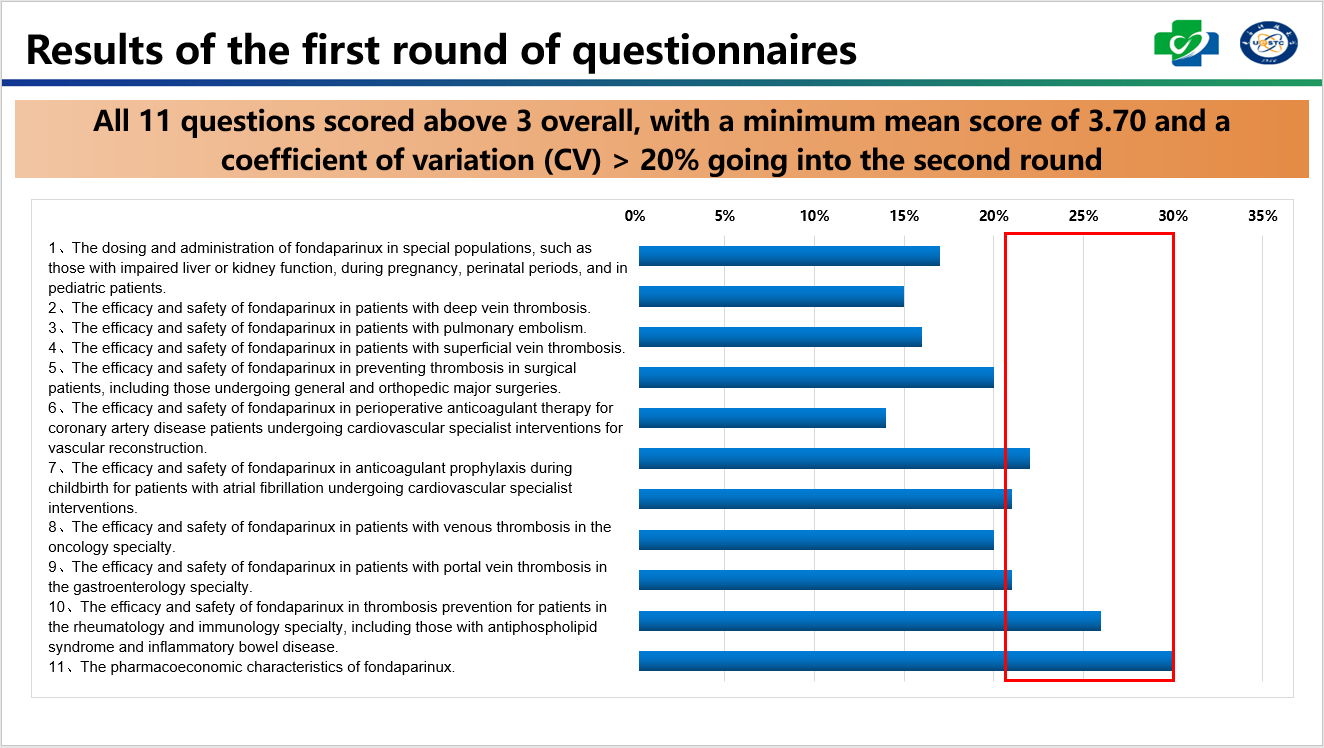
In the first round of the questionnaires, encompassing the 11 initially identified issues, the clinical issues exhibiting a notable disparity in scores (with a coefficient of variation > 20%) subsequent to evaluation by the experts were identified as **6, 7, 9, 10, and 11**. Consequently, a subsequent round of scoring was conducted for these five issues, newly added and/or any omitted clinical issues.


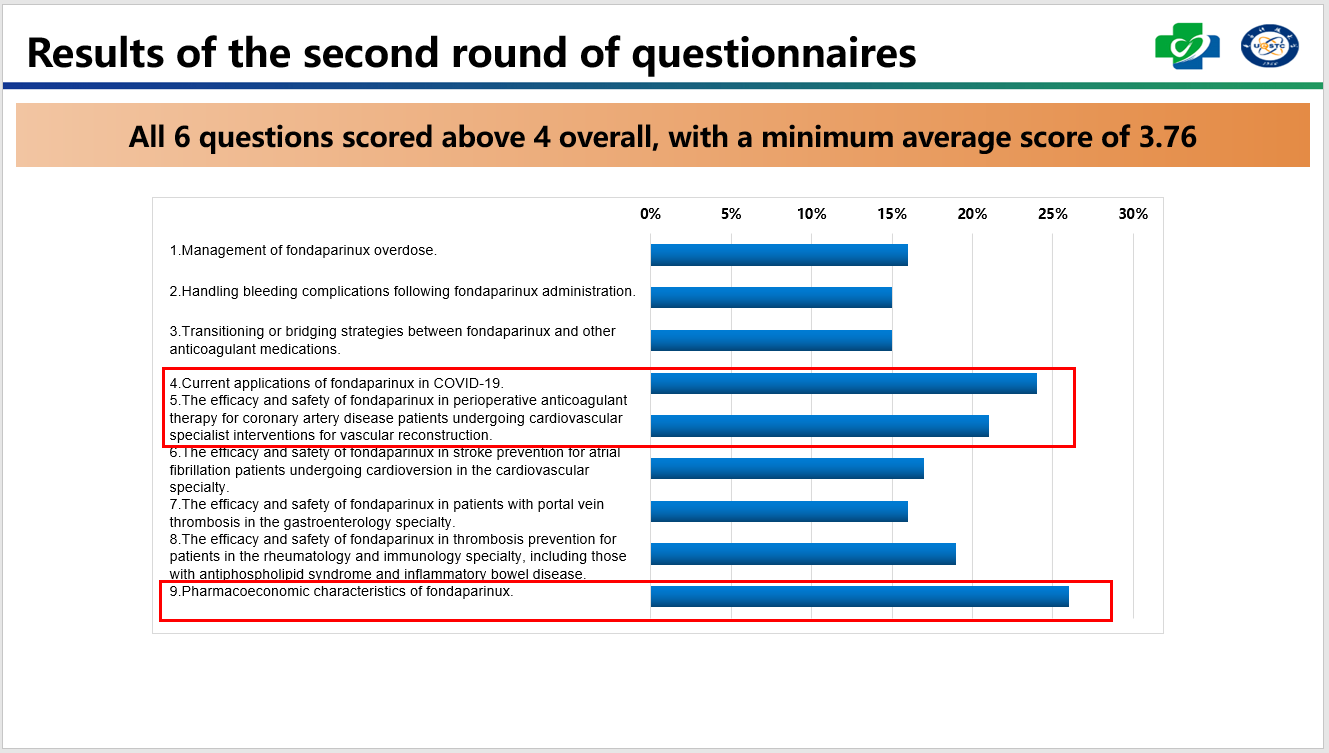
In the second round of the questionnaires, nine issues were included, of which the clinical issues that were scored by the experts with significant differences in the scores (coefficient of variation > 20%) were numbered **4, 5, and 9**, respectively. these three issues were therefore removed.


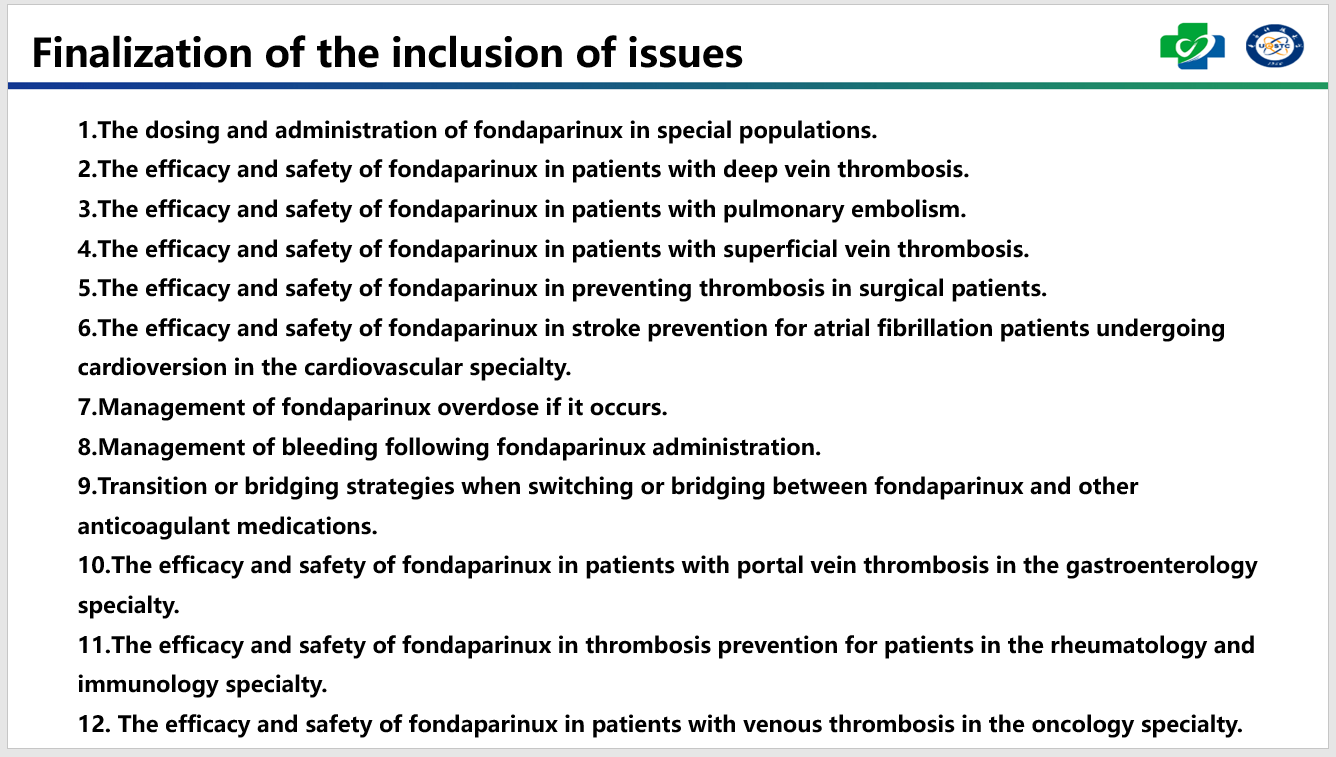
12 issues were eventually included.

**Part Ⅲ**

Recommendations that cover a number of clinical trials and statistical analyses greater than 5 have only recommendation 7.

Summary of Clinical Trials and Statistical Analyses in Recommendation 7

| **Author/Year** | **Type of study** | **Type of surgery** | **research subjects** | **Treatment** | **Results** |
| --- | --- | --- | --- | --- | --- |
| (Yang et al., 2023) | observational study | unilateral TKA | patients were divided into LMWH and fondaparinux groups (34 and 37 patients, respectively) | The LMWH group received low molecular weight heparin calcium injection subcutaneously at half the normal dose 4–6 h after the procedure. The normal dose was injected (one injection per day) on the following day. The fondaparinux group was subcutaneously injected with a 2.5 mg dose, starting at 6 h after surgery (one injection per day). The treatment duration was 2 weeks. | Within-group pairwise comparisons of preoperative, 1-day or 3-day postoperative d-dimer or fibrinogen levels showed significant differences (all p < 0.05). Between-group differences in preoperative prothrombin time, prothrombin time, activated fractional prothrombin time, and international normalized ratio were significant at days 1 and 3 postoperatively (all p < 0.05).  Pairwise comparisons of hemoglobin and hematocrit levels between the same groups of patients before and 1 or 3 days after surgery showed significant differences between the two groups (all p < 0.05). Within-group differences in visual analog scale scores before and 1 or 3 days after surgery were significant (p < 0.05). |
| (Fuji et al., 2008) | RCT | TKR or THR surgery  TKR or THR revision surgery | 339 in the fondaparinux groups  87 in the placebo group | Patients were assigned to receive a once-daily subcutaneous injection of fondaparinux (0.75 mg, 1.5 mg, 2.5 mg, or 3.0 mg) or placebo. | In the intent to treat population, 65.3%, 34.2%, 21.3%, 16.2% and 9.5% of the patients showed VTE in the groups given placebo, 0.75 mg, 1.5 mg, 2.5 mg, and 3.0 mg of fondaparinux respectively.  The calculated relative risk reductions of VTE with 0.75 mg, 1.5 mg, 2.5 mg and 3.0 mg of fondaparinux, compared with placebo, were 47.6%, 67.4%, 75.2%, and 85.5% respectively. |
|  |  |  | 324 in the fondaparinux groups  82 in the placebo group |  | In the intent to treat population, 33.8%, 24.2%, 4.6%, 7.4% and 14.3% of the patients showed VTE in the groups receiving placebo, 0.75 mg, 1.5 mg, 2.5 mg, and 3.0 mg of fondaparinux respectively.  Relative risk reductions of VTE with 0.75 mg, 1.5 mg, 2.5 mg and 3.0 mg of fondaparinux were 28.4%, 86.4%, 78.1%, and 57.7% respectively, compared with placebo. |
| (Li et al., 2013) | meta-analysis | major knee surgery  hip fracture surgery  THA | five RCTs  7611 patients. | - | The incidences of total VTE and DVT were significantly lower in fondaparinux group than in enoxaparin group (RR = 0.52,95%CI = 0.40-0.67, P < 0.00001; RR = 0.49, 95%CI = 0.42-0.58, P < 0.00001).  The incidence of symptomatic VTE was similar between the two groups (RR = 1.52,95%CI = 0.80-2.88, P = 0.201).  Fondaparinux was associated with a significantly increased incidence of major bleeding compared to enoxaparin group (RR = 1.55, 95%CI = 1.14-2.12, P = 0.006), but the mortality rates were comparable between the two groups (RR = 0.93, 95%CI = 0.63-1.37, P = 0.72). |
| (Migita et al., 2014) | observational study | TKA  THA  (University Hospital Medical Information Network Clinical Trials Registry: UMIN000001366) | 1,294 patients underwent total knee arthroplasty (TKA)  868 patients underwent total hip arthroplasty (THA) | - | Only prophylaxis with fondaparinux reduced the occurrence of VTE significantly in both groups. Propensity score matching analysis (fondaparinux vs. enoxaparin) showed that the incidence of DVT was lower (RR = 0.70, 95% CI = 0.58-0.85, P = 0.002 in TKA and relative risk 0.73, 95% CI = 0.53-0.99, P = 0.134 in THA) but that the incidence of major bleeding was higher in the fondaparinux than in the enoxaparin group (3.4% vs. 0.5%, P = 0.062 in TKA and 4.9% vs. 0%, P = 0.022 in THA). |
| (He et al., 2021) | meta-analysis | TKA  THA | Thirty-five RCTs 53787 patients | - | Fondaparinux (88.89% ± 10.90%) was ranked first for anticoagulant effectiveness. |

LMWH: low molecular weight heparin; RR: relative risk; CI: confidence interval; VTE: venous thromboembolism; DVT: deep vein thrombosis; TKA: total knee arthroplasty; THA: total hip arthroplasty; RCT: randomized controlled trial; TKR: total knee replacement; THR: total hip replacement

References

Fuji, T., Fujita, S., and Ochi, T. (2008). Fondaparinux prevents venous thromboembolism after joint replacement surgery in Japanese patients. *Int Orthop* 32(4)**,** 443-451. doi: 10.1007/s00264-007-0360-7.

He, T., Han, F., Wang, J., Hu, Y., and Zhu, J. (2021). Efficacy and safety of anticoagulants for postoperative thrombophylaxis in total hip and knee arthroplasty: A PRISMA-compliant Bayesian network meta-analysis. *PLoS One* 16(6)**,** e0250096. doi: 10.1371/journal.pone.0250096.

Li, H., Wang, J., Xiao, J., and Shi, Z. (2013). [Efficacy and safety of fondaparinux versus enoxaparin for preventing venous thromboembolism after major orthopedic surgery: a meta-analysis]. *Nan Fang Yi Ke Da Xue Xue Bao* 33(3)**,** 370-375.

Migita, K., Bito, S., Nakamura, M., Miyata, S., Saito, M., Kakizaki, H., et al. (2014). Venous thromboembolism after total joint arthroplasty: results from a Japanese multicenter cohort study. *Arthritis Res Ther* 16(4)**,** R154. doi: 10.1186/ar4616.

Yang, T., Liu, Z., Zhang, B., Zhang, J., Ma, A., Cao, D., et al. (2023). Comparison of the efficacy of low-molecular-weight heparin and fondaparinux sodium after total knee arthroplasty: a retrospective cohort study. *BMC Musculoskelet Disord* 24(1)**,** 552. doi: 10.1186/s12891-023-06674-6.
